# Systematic review of pregnancy and renal outcomes for women with chronic kidney disease receiving assisted reproductive therapy

**DOI:** 10.1007/s40620-022-01510-x

**Published:** 2022-11-18

**Authors:** Mahua Bhaduri, Rouvick M. Gama, T. Copeland, Alokya Balagamage, Priya Patel, Emily Warmington, Ippokratis Sarris, Kypros Nicholaides, Kate Bramham

**Affiliations:** 1 King’s Fertility, London, UK; 2 Department of Women and Children’s Health, King’s College London, London, UK; 3 King’s College London School of Medicine, London, UK; 4 College of Medicine and Dental Sciences, University of Birmingham, Birmingham, England; 5 Fetal Medicine Research Institute, Harris Birthright Centre, King’s College Hospital, London, UK

**Keywords:** Pregnancy, Fertility treatment, Chronic kidney disease, Fetal outcomes

## Abstract

**Background:**

As awareness around infertility is increasing among patients with chronic kidney disease (CKD), ever more of them are seeking Assisted Reproductive Technology (ART). Our aim was to perform a systematic review to describe obstetric and renal outcomes in women with CKD following ART.

**Methods:**

The following databases were searched from 1946 to May 2021: (1) Cochrane Central Register of Controlled Trials (CENTRAL), (2) Cumulative Index to Nursing and Allied Health Literature (CINAHL), (3) Embase and (4) MEDLINE.

**Results:**

The database search identified 3520 records, of which 32 publications were suitable. A total of 84 fertility treatment cycles were analysed in 68 women. Median age at time of pregnancy was 32.5 years (IQR 30.0, 33.9 years). There were 60 clinical pregnancies resulting in 70 live births (including 16 multifetal births). Four women developed ovarian hyperstimulation syndrome which were associated with acute kidney injury. Hypertensive disorders complicated 26 pregnancies (38.3%), 24 (35.3%) pregnancies were preterm delivery, and low birth weight was present in 42.6% of pregnancies. Rates of live birth and miscarriage were similar for women with CKD requiring ART or having natural conception. However, more women with ART developed pre-eclampsia (*p < *0.05) and had multifetal deliveries (*p < *0.001), furthermore the babies were lower gestational ages (*p < *0.001) and had lower birth weights (*p < *0.001).

**Conclusion:**

This systematic review represents the most comprehensive assessment of fertility outcomes in patients with CKD following ART. However, the high reported live birth rate is likely related to reporting bias. Patient selection remains crucial in order to maximise patient safety, screen for adverse events and optimise fertility outcomes.

**Supplementary Information:**

The online version contains supplementary material available at 10.1007/s40620-022-01510-x.

## Introduction

The prevalence of chronic kidney disease (CKD) in women of reproductive age continues to rise, and 15% of all affected individuals worldwide are diagnosed before the age of 50 [1]. However, pregnancy rates in women with CKD are low; women with end stage kidney disease (ESKD) make up 1% of the pregnancy rates reported in the general population [2]. Over recent decades, the number of women undergoing renal transplantation has risen resulting in a greater proportion becoming pregnant, albeit still at lower rates (10%) compared to the general population [2]. Individual choice, clinician influence and reduced fertility are all likely to have contributed to this [2].

Although precise mechanisms of how CKD reduces fertility are not completely understood, it is likely to be multifactorial. Contributory mechanisms include hypothalamus-pituitary-ovarian axis dysfunction, reduction in ovarian reserve and increased inflammation, all of which negatively impact on reproductive potential [2]. Furthermore, loss of pulsatile release of Gonadotropin releasing hormone (GnRH) impairs ovulation and so, as kidney function declines, women can experience loss of, or irregular, menstruation [3].

The use of assisted reproductive technologies (ARTs) has rapidly expanded since the 1980s, resulting in over 8 million live births [4]. ARTs include ovulation induction (OI), intrauterine insemination (IUI), in vitro fertilization (IVF) and intracytoplasmic sperm injection (ICSI). OI involves selective oestrogen-receptor modulation, usually with oral clomiphene or letrozole, which increases follicle-stimulating hormone (FSH) release. This is typically given in the first part of the cycle to stimulate one or two dominant follicles which will ovulate [5]. It can be used in conjunction with IUI, where sperm is prepared and injected into the uterus at the optimal time for fertilisation [5]. IVF requires high concentrations of FSH being administered to the patient, increasing the size of the ovaries before the oocytes are collected from the ovaries via a needle passed through the vaginal wall and fertilised with sperm in vitro [6]. Common risks of the procedure include post-procedure pain and minor bleeding, much rarer complications include damage to surrounding structures and infection [7].

Although fertility treatment is generally very safe, there are risks and potential complications. Ovarian hyperstimulation syndrome (OHSS) is a potential, serious complication of fertility treatment caused by over response to gonadotropin intake. It is characterised by cystic enlargement of the ovaries and a fluid shift from the intravascular to the third space due to increased capillary permeability and ovarian neoangiogenesis [8]. Mild OHSS includes symptoms such as abdominal discomfort and nausea, whereas severe cases can lead to acute renal failure, venous thrombosis and pulmonary oedema. The incidence of moderate OHSS is 3–6%, whereas severe OHSS occurs in 0.1–3% of patients [8].

Current family planning trends show that women are delaying pregnancies until their late 30 s early-mid 40 s*,*[9], as demonstrated by the rate of live births to mothers aged 35 and over rising from 8.7% in 1990 to 20% in 2012 [10]. Hence, infertility secondary to advanced age has led to more people exploring ARTs.

As the quality and quantity of life in CKD patients has improved with advanced medicine, more CKD patients are seeking fertility treatment to assist them. The first reported live birth following IVF in a renal transplant patient was in 1995 [11]. Since then, there have been a growing number of documented cases of CKD patients having successful fertility treatment [12]. However, both IVF and CKD are independently associated with increased risk of preterm birth, low birth weight and perinatal mortality [12, 13]. Therefore concerns remain about maternal and fetal safety with fertility treatment in women with CKD as well as the potential detrimental impact on kidney function.

Our primary aim was to perform a systematic review to describe pregnancy and kidney outcomes and complications of pregnancies in women with CKD following fertility treatment. Our secondary aims were to compare the outcomes of CKD patients undergoing ART both to those with natural conception and to those without CKD.

## Methods

The systematic review was performed with reference to the Cochrane Handbook for Systematic Reviews of Interventions and reported with reference to the Preferred Reporting Items for Systematic Reviews and Meta-Analyses (PRISMA) guidelines [14, 15]. The protocol was registered on PROSPERO: International Prospective Register of Systematic Review (CRD42021254861; www.crd.york.ac.uk/PROSPERO).

### Search strategy

The following databases were searched from 1946 to May 2021: (1) Cochrane Central Register of Controlled Trials (CENTRAL), (2) Cumulative Index to Nursing and Allied Health Literature (CINAHL), (3) Embase and (4) MEDLINE for MeSH headings: (1) Chronic kidney disease; (2) Fertility treatment; (3) infertility therapy (Supplementary material 1). Relevant review articles were also searched for additional studies.

### Selection criteria

Five researchers (MB, RG, AB, PP and EW) independently screened titles and abstracts and full text of selected studies to assess eligibility. Any discrepancies between the reviewers were resolved by a further senior researcher (KB). There was good agreement between reviewers, and discrepancies were easily resolved. The kappa agreement between the reviewers was 0.81 showing almost perfect agreement. All published case reports, case series, and retrospective analyses of chronic kidney disease patients with fertility treatment were evaluated. Studies were excluded if they were only abstracts, not available in English, did not assess fertility outcomes or did not include patients with CKD or used surrogates to carry the pregnancy.

### Quality of the included studies

The quality of our study was assessed independently by two authors (MB, RG) according to the Joanna Briggs Institute (JBI) Critical Appraisal Tools for Case reports/ Case series [16]. Disagreement between the two authors were resolved with the help of a senior researcher (KB).

A scoring system was used to rate the quality of included studies, with scores of 7 or greater being given for ‘High’ quality assessment, 4–6 for ‘Medium’ and < 4 for ‘Low’. The scores for each publication are detailed in Table 1.

**Table 1:** Studies included in our analysis with outcomes and quality assessment

	Reference, year published	Country	Number of patients	Pregnancies	Quality Assessment
1	Wei et al. 2019	China	1	1	Moderate
2	Gramkow et al. 2014	Denmark	1	1	Moderate
3	Piccoli et al. 2013	Italy	5	5	Moderate
4	Loeffler 2005	USA	1	1	Low
5	Chehab 2019	Germany	1	1	Moderate
6	Nicovani et al. 2009	Chile	1	1	High
7	Furman et al. 1999	Israel	2	2	High
8	Kovacs et al. 2015	Hungry	2	1	Low
9	Huong et al. 2001	France	2	2	Low
10	Mahmoud et al. 2017	Turkey	1	1	High
11	Lockwood et al. 1995	UK	1	1	High
12	Mishra et al. 2016	India	3	0	Moderate
13	Muthuvel et al. 2016	Unknown	1	1	High
14	Zheng et al. 2016	China	1	0	High
15	Nouri et al. 2010	Austria	2	2	Moderate
16	Kennedy et al. 2012	Ireland	1	1	NA
17	Warzecha et al. 2018	Poland	4	4	High
18	Motoyama et al. 2018	Japan	1	1	High
19	Tamaki et al. 2003	Japan	1	1	Moderate
20	Moyer et al. 1996	USA	1	1	Moderate
22	Khalaf et al. 2000	UK	1	1	High
23	Yuksel et al. 2017	Turkey	3	3	Moderate
24	Bateman et al. 2019	Australia	2	1	Moderate
25	Yaprak et al. 2019	Turkey	13	11	Moderate
27	Morisawa et al. 2019	Japan	1	1	Low
28	Rao et al. 2018	Australia	1	1	High
29	Kosoku et al. 2019	Japan	1	1	High
30	Mohammadi et al. 2018	Australia	1	1	High
31	Pietrzak et al. 2015	Poland	1	1	High
33	Fichez et al. 2008	France	1	1	High
34	Choi et al. 2018	Korea	1	0	Moderate
35	Normann et al. 2014	Sweden	8	8	High

### Data extraction

Two researchers (MB and RG) extracted all relevant data for each study independently using a pilot‐tested data extraction sheet using a minimum core outcome data set developed for fertility research using formal consensus methods, [15] including primary outcome, live birth; and secondary outcomes: (1) clinical pregnancy, (2) pregnancy loss due to miscarriage, ectopic pregnancy, stillbirth, (3) gestational age at delivery, (4) birthweight, (5) neonatal mortality and (6) major congenital anomalies, (7) renal complications (acute kidney injury and requiring renal replacement therapy) and (8) obstetric complications [17].

Maternal age at time of pregnancy (years), renal data (underlying cause and stage of CKD, type of renal replacement therapy (RRT), serum creatinine concentration, stage of acute kidney injury (AKI) as per Kidney Disease: Improving Global Outcomes (KDIGO) criteria), [18] comorbidities and medications were independently extracted. Immunosuppressive medications including changes to therapy in pregnancy, long-standing drug history and fertility treatments were all recorded. AKI stage was calculated, when not provided by the authors, using change in serum creatinine, as per KDIGO criteria [19]. CKD stage was calculated using The Chronic Kidney Disease Epidemiology Collaboration (CKD-EPI) eGFR equation from reported pre-pregnancy serum creatinine concentrations [20]. Cause of infertility, type of ART used and mode of delivery were also extracted.

Authors were contacted to seek clarification and to request missing data or additional data to complete our analysis. Outcomes were compared for 60 pregnancies in women with CKD with ART against 504 pregnancies in women with CKD without ART [21].

### Statistical analysis

Data are presented as counts with percentages, mean ± SD and range for parametric data and median with interquartile range for non-parametric data. The data collected were compared to those reported in previous literature of patients with CKD who had had pregnancy via natural conception. Statistical significance was assessed using Chi-squared test for non-parametric data with a *p* value < 0.05 as significant.

## Results

**Fig. 1:**
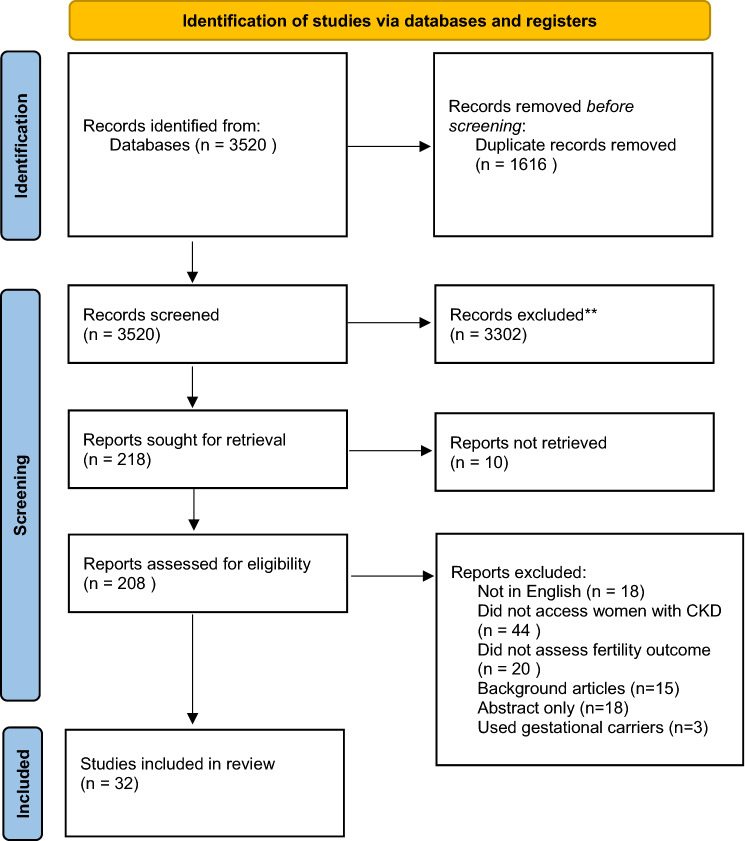
Study flow diagram

An initial search of the online databases identified 3520 records of which 1616 were duplicates. Titles and abstracts of the remaining 1904 records published between 1995 and 2020 were screened, leading to 32 publications being quantitatively analysed (Fig. 1) including 23 case reports, three case series, four retrospective studies, one population-based retrospective study and 1 questionnaire.

There were 68 women for whom a total of 84 fertility treatment cycles were analysed (35 women with data extracted from case reports or series, 32 from retrospective studies and 1 from other publication types). Fertility treatment cycles included IVF/ICSI cycles, OI cycles, IUI cycles, frozen embryo transfer cycles and auto-transplantation of cryopreserved ovarian tissue.

### Demographic and baseline characteristics

The median age of women with CKD at the time of pregnancy was 32.5 years (IQR 30.0, 33.9 years; Range 25–45 years). Ethnicity was not reported in any of the studies. Clinical characteristics are described in Table 2.

**Table 2:** Demographic and clinical data of CKD women who had fertility treatment

	Women with CKD (*n = *68)
Median Age of women at pregnancy (IQR), years	32.5 (30.0, 33.9)
Cause of renal disease (*n* (%))	
CPN/VUR	9 (13.2%)
Glomerulonephritis	19 (27.9%)
SLE	5 (7.4%)
ADPKD	5 (7.4%)
Diabetes	5 (7.4%)
Congenital/inherited	6 (8.8%)
Other	6 (8.8%)
Unknown	13 (19.1%)
Co-morbidities, *n = *22 (*n* (%))	
Hypertension	10 (19.2%)
Diabetes mellitus	4 (7.7%)
Hepatitis B	2 (3.8%)
Hepatitis C	2 (3.8%)
Crohn’s disease	1 (1.9%)
Turner’s syndrome	1 (1.9%)
Anaemia	1 (1.9%)
Recurrent UTIs	1 (1.9%)
Native Kidney CKD stage (*n* (%))	
Stage 1	6 (37.5%)
Stage 2	2 (12.5%)
Stage 3	0 (0%)
Stage 4	0 (0%)
ESKD-HD	1 (6.25%)
ESKD-PD	1 (6.25%)
Unknown	6 (37.5%)
Kidney transplant	52 (76.4%)
Transplant vintage at time of fertility treatment (years (IQR)) (*n = *52)	4.5 (4.0, 7.0)
Pre-pregnancy immunosuppressive drugs	
Prednisolone/methylprednisolone	40 (76.9%)
Azathioprine	22 (42.3%)
Cyclosporine	27 (51.9%)
Mycophenolate mofetil	5 (9.6%)
Tacrolimus	23 (44.2%)
Rituximab	1 (1.9%)
Aspirin	4 (5.4%)
Number of immunosuppressive drugs (*n = *66) (*n* (%))	
3	24 (46.2%)
2	16 (30.8%)
1	9 (17.3%)

*CPN* chronic pyelonephritis; *VUR* vesicoureteral reflux; *SLE* systemic lupus erythematosus; *ADPKD* autosomal dominant polycystic kidney disease, *ESKD-HD* end stage kidney disease-haemodialysis, *ESKD-PD* end stage kidney disease- peritoneal dialysis

### Renal data and outcomes

A substantial proportion of women had renal transplants (*n = *52, 76.5%) with a median time from transplantation to fertility treatment of 4.5 years (IQR 4.0, 7.0 years). For women without transplants (*n = *16), eight had early CKD (Stage 1 = 6, Stage 2 = 2) and two patients were established on dialysis prior to conception.

The pre-pregnancy, median serum creatinine (*N = *30) was 88 µmol/L (IQR 71, 106 µmol/L); first trimester (*N = *4) 115 µmol/L (IQR 97, 133 µmol/L), second trimester (*N = *10) 69 µmol/L (IQR 49, 106 µmol/L), third trimester (*N = *16) 113 µmol/L (IQR 84, 164) and post-partum (*N = *4) 168 µmol/L (IQR 137, 198 µmol/L). Median serum creatinine levels before, during and after pregnancy are summarised in Table 3.

**Table 3:** Median serum creatinine and eGFR in each pregnancy trimester

All	Median serum creatinine (IQR), µmol/L	CKD-EPI eGFR (IQR), mL/min/1.73m^2^
Pre-pregnancy Baseline (*N = *26)	88 (71, 106)	75 (61, 99)
1st Trimester (*N = *3)	115 (97, 133)	56 (48, 72)
2nd Trimester (*N = *8)	69 (49, 106)	102 (62, 122)
3rd Trimester (*N = *16)	113 (84, 164)	57 (38, 82)
Post-partum (*N = *4)	168 (137, 198)	36 (31, 52)
Transplant		
Pre-pregnancy baseline (*N = *18)	96 (88, 121)	67 (51, 76)
1st Trimester (*N = *2)	133 (124, 142)	48 (44, 52)
2nd Trimester (*N = *3)	106 (91, 142)	62 (48, 76)
3rd Trimester (*N = *10)	141 (108, 172)	44 (35, 60)
Post-partum (*N = *3)	159 (115, 168)	37 (36, 67)
Native kidneys		
Pre-pregnancy baseline (*N = *8)	62 (48, 76)	104 (90, 124)
1st Trimester (*N = *1)	80	88
2nd Trimester (*N = *6)	52 (45, 65)	117 (105, 127)
3rd Trimester (*N = *6)	72 (56, 93)	93 (72, 117)
Post-partum (*N = *1)	264	21

The overall pre-pregnancy median estimated glomerular filtration rate (eGFR) was 75.0 ml/min/1.73m^2^ (IQR 61, 99 ml/min/1.73m^2^) and it was 67 ml/min/1.73m^2^ (IQR 51, 76.0 ml/min/1.73m^2^) in women with kidney transplants (*N = *30) and 99.0 ml/min/1.73m^2^ (IQR 86.0, 120.0 ml/min/1.73m^2^) in women with CKD without transplants (*N = *9). Ten women who reported creatinine changes met criteria for AKI (6 – Stage 1, 1-Stage 2; 3- Stage 3). All stage 3 AKI episodes required emergency haemodialysis / haemofiltration. Factors contributing to AKI included atypical haemolytic uraemic syndrome (aHUS), non-aHUS thrombotic microangiopathy, nephrotic syndrome relapse, pre-eclampsia (*N = *5), OHSS (*N = *2) and unknown (*N = *2).

Women were also taking many other medications including antihypertensives (nifedipine, labetalol, metoprolol, losartan, quinapril, methyldopa), aspirin, acyclovir, co-amilozide, insulin, calcitriol, omeprazole, atorvastatin and erythropoietin including teratogenic co-trimoxazole. The causes of CKD for women undergoing fertility treatment, and the immunosuppressive medications taken by women with kidney transplants (*N = *52) are listed in Table 2.

### Type and cause of infertility, fertility treatment and outcomes

The cause of infertility was reported for 28 out of 68 women (41.2%). Of those reported, the most common cause was tubal (*n = *7, 25%). Anovulatory disorders occurred in 6 women (21.4%) including polycystic ovary syndrome. Secondary infertility was identified in 5 women (17.9%). Male factor was identified in 2 cases (10.7%). Other causes included primary ovarian failure / premature ovarian insufficiency (*n = *2, 7.1%), endometriosis (*n = *2, 7.1%), low ovarian reserve (*n = *1, 3.6%), genetic abnormalities (*n = *1, 3.6%) and 1 reported as unexplained (3.6%).

The majority of women had IVF or ICSI (54/68; 79.4%). One woman had an auto-transplant of cryopreserved ovarian tissue which led to a spontaneous pregnancy, one woman had a medicated frozen embryo transfer cycle after a previous IVF cycle to create the transferred embryo, one woman used donor eggs, seven women (10.1%) had OI and two women had IUI (2.9%).

OI regimens varied, most commonly using clomiphene citrate and human menopausal gondatropin with many different protocols used for IVF mid-luteal pituitary downregulation and antagonist approaches. Controlled ovarian stimulation medications included Follitropin alfa, follitropin beta, urofollitropin, Human Chorionic Gonadotropin (HCG), gonadorelin analogues and LHRH agonist triggers. When reported, luteal support was given in intramuscular, vaginal and rectal forms. The median duration of controlled ovarian stimulation was 11 days (IQR 10.5, 11 days).

Fertility and obstetric outcomes are summarised in Table 4. The number of embryos transferred was unknown in 27 women, and the reported number of embryos ranged from one to six. In 12 cases a single embryo was transferred, and a double embryo transfer was reported in 12 cases.

**Table 4:** Fertility and pregnancy outcomes of Chronic Kidney Disease (CKD) patients receiving Assisted Reproductive Technology (ART) treatment compared with cohorts of women with CKD with spontaneous conception and women with kidney transplant who had spontaneous pregnancies and women who had ART following kidney transplantation

	Women with CKD with ART (16 women)	Women with CKD with spontaneous pregnancy(21)	*p* values	Women with kidney transplant with ART (52 women)	Women with kidney transplant with spontaneous pregnancy(22)	*p* values
Live births, % (*n*): pregnancy resulting in live birth	86.7% (13/15)	68.9% (504/731)	0.141	84.4% (38/45)	76.3% (5120/6712)	0.199
Miscarriages %, (*n*)	13.3% (2/15)	4.9% (36/731)	0.143	11.1% (5/45)	15.4%	0.426
Stillbirths/ neonatal death, % (*n*)	0% (0/15)	0.55% (4/731)	0.774	4.4% (2/45)	5.1%	0.843
Ectopic pregnancies, % (*n*)	0% (0/15)	0	N/A	2.2% (1/45)	2.4%	< 0.001
Multiple birth rate, % (*n*)	60% (9/15)	3.4%, (25/731)	< 0.001	15.6% (7/45)	–	
IVF complications per IVF cycle OHSS (*n*, %)	1/20, 5%	–		3/64, 4.7%	–	
Live births (*n*)	23 fetuses			47 fetuses		
Median gestational age (IQR), weeks + days	28 + 2 (28 + 0, 32 + 4)			34 + 2 (32 + 2, 37 + 4 days		
Mean gestational age (weeks)	27.6 ± 9.3	36.9 ± 2.9	< 0.001	32.2 ± 7.7	34.7 weeks ± 2.25	< 0.001
Median birth weight (IQR), g	1540 (1013, 1980)	-		1980 (1311, 2615)		
Mean singleton birth weight, g	2319 ± 255.972655	2802.6 ± 728	0.0172	2014 ± 689	2473.7 ± 251.9	< 0.001
Median singleton birth weight (IQR), g	2319 (2229, 2410)			2322 (1810, 2666)		
Low (< 2500 g), (%)	18/19, 94.7%			20/47,42.6%		
Very low (< 1500 g), (%)	9/19, 47.3%			15/47, 31.9%		
Caesarean section, *n* (%)	7/15, 46.7%	54.8%		34/38, 89.5%	62.6%	
Unknown mode of delivery, *n* (%)	8/15(42.4%)			0/38		

**Fig. 2:**
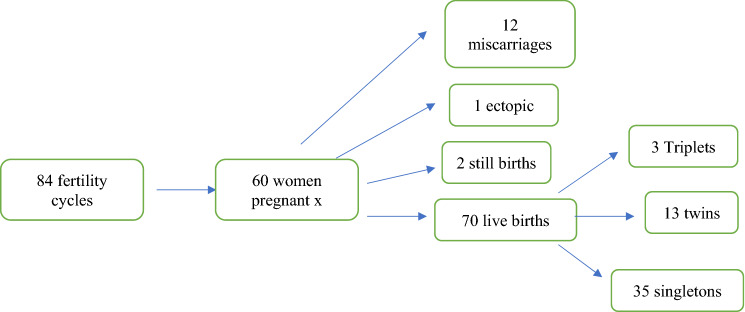
Overview of fertility cycles, pregnancies and fetal/neonatal outcome numbers

There were 84 fertility cycles from 68 patients. The overall pregnancy rate per fertility cycle was 60/84 (71.4%) and live birth rate of 51/60 (85%). Live birth rate was determined as the number of pregnancies which ended with at least 1 live birth. In total there were 70 babies: there were 13 sets of twins, including a triplet pregnancy that was reduced to twins and a quintruple pregnancy that was reduced to twins. There were 3 sets of triplets including a quadruplet pregnancy that was reduced to triplets. There were 35 singletons borne including 2 sets of twins which reduced to singletons spontaneously. Of the pregnancies that did not continue to live birth: there were 12 miscarriages, 1 ectopic pregnancy and 2 stillbirths. This is summarised in Fig. 2.

Four women (4/54; 7.4%) developed Ovarian Hyperstimulation Syndrome, three of whom developed AKI. One woman also developed pancreatitis and OHSS three weeks after embryo transfer and was successfully treated with intravenous fluids, methylprednisolone, heparin and albumin.

### Obstetric outcomes

Twenty-six/68 (38.3%) women were complicated by hypertensive disorders of pregnancy including pre-eclampsia (*N = *18; 26.5%) and gestational hypertension (*N = *8; 11.8%). Other complications included obstetric cholestasis (*N = *1), anaemia (*N = *3), DVT (*N = *1) and urinary tract infection (*N = *1). The most common mode of delivery was caesarean Sect. (41/68; 60.3%). Four (5.9%) women had vaginal deliveries and mode of delivery was not reported for 23/68 (33.8%).

Preterm delivery (< 37 weeks’ gestation) occurred in 24/68 (35.3%) women. Overall median gestational age of delivery was 34 weeks (IQR 30, 36) and median birth weight was 1658 g (IQR 1190, 2295); Singletons only: 2322 g (IQR 1812, 2649). Median intergrowth centile for singletons was 55.8 (IQR 15.1, 60.5), 23.5 (IQR 11.7, 23.7) for twins and 12.0 (IQR 16.8, 39.9) for triplets. Low birth weight (< 2500 g) was present in 29/68 (42.6%) of women.

### Fetal and long-term outcomes

There were 14 neonate admissions to a neonatal intensive care unit with median stay of 52.5 days (IQR 16.5, 81.5). Five out of 70 (7.1%) neonates had congenital malformations which included an ear malformation, inguinal and umbilical hernias, hydrocele and phimosis. Fourteen offspring were followed up for up to four years. One child developed hyperactivity disorder.

### Comparison between the outcomes in the renal transplantation cohort and non-transplant group

We further subdivided the fertility and pregnancy outcomes of the patients according to transplant status as shown in Table 4. In addition, we compared the outcomes with previous literature published on spontaneous pregnancies in patients with CKD and patients who had had spontaneous pregnancies with renal transplant [21, 22].

The majority of the women in our cohort had had renal transplant ( 52 women). Only 16 women had not had renal transplant, of whom 15 had pregnancies. When comparing the non-transplant patients who had ART with non-transplant patients with spontaneous pregnancies we observed that the live birth rate (86.7% vs 68.9%) and miscarriage rate (13.3% vs 4.9%) were not significantly different but the multiple pregnancy rate (60% vs 3.4%) in the ART group was significantly higher (*p < *0.001) [21]. The rate of pre-eclampsia in the ART CKD cohort (6/15, 37.5%) was much higher than the spontaneous pregnancy CKD cohort (95/731, 13.0%) (*p < *0.002). The mean gestational age was lower in the ART CKD women ( 27.6 weeks, cf. 36.9 weeks) as was the mean singleton birth weight (2319 g, cf. 2803 g) which were both statistically significant (*p < *0.05) [21].

There were 52 women who had ART after having a kidney transplant. This cohort was compared to women with kidney transplant with spontaneous pregnancy and is summarised in Table 4 [22]. The live birth rate and miscarriage rate (11.1% cf 15.4%) were similar as was the stillbirth rate. The mean gestational age was significantly lower in the ART cohort compared to the spontaneous pregnancy cohort (32.2 weeks cf 34.9 weeks) (*p < *0.001)and the mean singleton birth weight was also significantly lower at 2014 g compared to 2470 g (*p < *0.001) [22].

## Discussion

To our knowledge, this systematic review represents the most comprehensive report describing fertility outcomes in patients with CKD who have assisted conception. Despite increasing requirement, there remains insufficient data reporting outcomes of fertility treatment in chronic disease to inform clinical care.

Reporting bias is likely to have confounded our findings, however our review of 84 cycles demonstrates that ART was generally well tolerated in patients with CKD, showing a high live birth rate of 85%. None of the reports described severe adverse events from fertility medications; only 4.8% of patients had complications from their fertility treatment and four women developed OHSS which was associated with AKI.

The live birth rate after fertility treatment was twice as high in our cohort than reported in healthy populations undergoing fertility treatment. The average birth rate per transferred embryo in the UK is 23%, [23] compared to 57.4% in the women in our cohort. Again, reporting bias is likely to have influenced this finding, but the high birth rate may be partly explained by the young age of women with CKD (median age of 32.5 years) compared with the IVF general population (median age is 35.8) [23].

Although most pregnancies resulted in live birth, fetal and maternal adverse outcomes were common. More than a quarter (26.5%) of women with CKD and ART developed preeclampsia and had a preterm delivery, which is seven-fold higher compared to the general USA population (35.3% vs. 3.8%) [18]. The rate of pre-eclampsia (p = 0.001) was significantly higher (*p < *0.05) in the ART CKD cohort when compared to women with CKD who had conceived naturally. However, it is also noted that patients who developed pre-eclampsia had lower eGFRs, hence this may have been a contributing factor as well as possibly ART.

Whilst there are no anatomical requirements for mode of delivery for women with CKD, even with kidney transplants, caesarean section rate for women with CKD and ART was approximately twice the rate of the general population receiving ART (60.3%) but similar to previous reports of CKD naturally conceived pregnancies [21, 22, 24, 31]. Fetal and maternal complications are commonly cited reasons for iatrogenic delivery in pregnant women with renal transplants without ART [26]. A recent Swedish national cohort study demonstrated that in 25.9% of CKD patients with chronic hypertension, singleton pregnancies had a medically indicated preterm birth (< 37 weeks) [24]. Preterm (< 37 weeks) and early preterm birth rates (< 34 weeks) were significantly higher in the ART CKD group than in the general population receiving ART as well as in CKD via natural conception, [19].

High preterm delivery rates could also be attributed to the rates of multifetal pregnancies (26.7%) in our cohort. It is well-known that increased maternal age and ART have increased the incidence of twin pregnancy by > 50%, but most recent Human Fertilisation and Embryology Authority reports suggest that multi-fetal pregnancies represent only 6% of ART pregnancies [23]. In the last decade, there has been an increasing drive towards single embryo transfer, especially in the UK and USA, which has led to a steep reduction in higher-order pregnancies.

Low birthweight (< 2500 g) was also high in women with CKD with ART compared to the general population receiving ART (8.7%), likely attributable to higher preterm birth and multifetal pregnancies. Interestingly, the live birth, miscarriage and stillbirth rates of women who had ART pregnancies living with renal transplants were similar to women with renal transplants with spontaneous pregnancies. However, birth weight and gestational age was significantly lower in the ART cohort which could be attributed again to the high multifetal pregnancy rate.

Assessment of renal function in women with CKD having ART was challenging due to insufficient data presented. However, in keeping with other pregnancies, both in women with and without CKD, there was a trend to a drop in serum creatinine in the second trimester and an increase post-partum, including for women with renal transplants [27, 28].

The majority of medications administered to women with CKD receiving ART had confirmed safety profiles in pregnancy, but in five cases mycophenolate mofetil was not stopped despite the clinicians actively trying to help their patients conceive. We did not find any reports of adverse incidents from any fertility medication used but none of the reports commented on whether adjustments in medication were made for women with CKD, and the diverse schedules reported prevent any robust assessment of optimal treatments.

The major limitation of our review is the heterogeneity and paucity of data, which were restricted to case reports, single-centre studies and voluntary registries which are inherently confounded by reporting bias and under-reporting, respectively [29, 30]. In addition, fertility outcomes over two decades were included, but in the last decade ART practice has changed considerably including the common practice of single embryo transfer which has led to improved maternal and neonatal outcomes which is not reflected in our review.

In conclusion, women with CKD appear to be able to undergo successful fertility treatment, with comparable pregnancy outcomes to women with CKD with natural conception; but prospective data collection is required to perform a robust assessment of the impact of CKD on ART outcomes, and the impact of ART on pregnancy outcomes in women with CKD, including optimal approaches to fertility treatments.

## Supplementary Information

Below is the link to the electronic supplementary material.

Supplementary file1 (DOCX 24 kb)

## Data Availability

The authors confirm that the data supporting the findings of this study are available within the article.

## References

[1] Centres for Disease Control and Prevention: Chronic kidney disease (CKD) Surveillance system. Prevalence and Incidence (2022). https://nccd.cdc.gov/ckd/TopicHome/PrevalenceIncidence.asp. Accessed 07 Mar 2022

[2] Wiles KS, Nelson-Piercy C, Bramham K (2018). Reproductive health and pregnancy in women with chronic kidney disease. Nat Rev Nephrol.

[3] Holley JL, Schmidt RJ, Bender FH (1997). Gynecologic and reproductive issues in women on dialysis. Am J Kidney Dis.

[4] Fauser BC (2019). Towards the global coverage of a unified registry of IVF outcomes. Reprod BioMed Online.

[5] Carson S, Kallen A (2021). Diagnosis and management of infertility. JAMA.

[6] Farquhar CM, Bhattacharya S, Repping S (2019). Female subfertility. Nat Rev Dis Primers.

[7] El-Shawarby S, Margara R, Trew G, Lavery S (2004). A review of complications following transvaginal oocyte retrieval for in-vitro fertilization. Hum Fertil.

[8] Royal College of Obstetrics and Gynaecologists: RCOG Green-top Guideline No. 5. The Management of Ovarian Hyperstimulation Syndrome (2022). https://www.rcog.org.uk/media/or1jqxbf/gtg_5_ohss.pdf. Accessed 17 July 2022

[9] Hayat MJ, Howlader N, Reichman ME, Edwards BK (2007). Cancer statistics, trends, and multiple primary cancer analyses from the surveillance, epidemiology, and end results (SEER) program. Oncologist.

[10] Kenny LC, Lavender T, McNamee R, O’Neill SM, Mills T, Khashan AS (2013). Advanced maternal age and adverse pregnancy outcome: evidence from a large contemporary cohort. PLoS ONE.

[11] Lockwood GM, Ledger WL, Barlow DH (1995). Successful pregnancy outcome in a renal transplant patient following in-vitro fertilization. Hum Reprod.

[12] Norrman E, Bergh C, Wennerholm U-B (2015). Pregnancy outcome and long-term follow-up after in vitro fertilization in women with renal transplantation. Hum Reproduct.

[13] Bergh C, Wennerholm U-B (2020). Long-term health of children conceived after assisted reproductive technology. Ups J Med Sci.

[14] Liberati A, Altman DG, Tetzlaff J (2009). The PRISMA statement for reporting systematic reviews and meta-analyses of studies that evaluate healthcare interventions: explanation and elaboration. BMJ.

[15] Higgins JP, Thomas J, Chandler J et al (2022) Cochrane Handbook for Systematic Reviews of Interventions version 6.3 (updated February 2022). Cochrane. www.training.cochrane.org/handbook. Accessed 07 Mar 2022

[16] Moola S, Munn Z, Tufanaru C et al (2017) Joanna Briggs Institute Reviewer's Manual. Chapter 7: Systematic reviews of etiology and risk. The Joanna Briggs Institute. https://reviewersmanual.joannabriggs.org/

[17] Duffy JMN, AlAhwany H, Bhattacharya S (2020). Developing a core outcome set for future infertility research: an international consensus development study. Hum Reprod.

[18] Zegers-Hochschild F, Adamson GD, Dyer S (2017). The International Glossary on Infertility and Fertility Care, 2017. Fertil Steril.

[19] KDIGO (2012) KDIGO: AKI. Kidney Int***

[20] Levey AS, Stevens LA, Schmid CH (2009). A new equation to estimate glomerular filtration rate. Ann Intern Med.

[21] Piccoli GB, Cabiddu G, Attini R (2015). Risk of adverse pregnancy outcomes in women with CKD. J Am Soc Nephrol.

[22] Shah S, Venkatesan RL, Gupta A et al (2019) Pregnancy outcomes in women with kidney transplant: Metaanalysis and systematic review. BMC Nephrol*** 10.1186/s12882-019-1213-5 PMC6345071 30674290

[23] Human Fertilisation and Embryology Authority: Fertility treatment 2018: trends and figures. UK statistics for IVF and DI treatment, storage, and donation (2022). https://www.hfea.gov.uk/about-us/publications/research-and-data/fertility-treatment-2018-trends-and-figures/. Accessed 07 Mar 2022

[24] Al-Khalaf SY, O’Reilly ÉJ, McCarthy FP (2021). Pregnancy outcomes in women with chronic kidney disease and chronic hypertension: a National cohort study. Am J Obstet Gynecol.

[25] Bramham K, Nelson-Piercy C, Gao H (2013). Pregnancy in renal transplant recipients: a UK National Cohort Study. Clin J Am Soc Nephrol.

[26] Deshpande NA, James NT, Kucirka LM (2011). Pregnancy outcomes in kidney transplant recipients: a systematic review and meta-analysis. Am J Transpl.

[27] Harel Z, McArthur E, Hladunewich M (2019). Serum Creatinine Levels Before, During, and after Pregnancy. J Am Med Assoc.

[28] Wiles K, Webster P, Seed PT (2021). The impact of chronic kidney disease Stages 3–5 on pregnancy outcomes. Nephrol Dial Transpl.

[29] Rizzoni G, Ehrich JHH, Broyer M (1992). Successful pregnancies in women on renal replacement therapy: Report from the EDTA Registry. Nephrol Dial Transpl.

[30] Davison JM, Redman CWG (1997). Pregnancy post-transplant: the establishment of a UK registry. BJOG.

[31] Poikkeus P, Gissler M, Unkila-Kallio L, Hyden-Gramskog C, Tiitinen A (2007). Obstetric and neonatal outcome after single embryo transfer. Hum Reprod.

